# Cutaneous Horn: A Masquerade to Underlying Keratotic Basal Cell Carcinoma

**DOI:** 10.7759/cureus.32427

**Published:** 2022-12-12

**Authors:** Khushdeep Abhaypal, Nazia Anjum, Manpreet Singh, Manu Saini, Pankaj Gupta

**Affiliations:** 1 Department of Ophthalmology, Post Graduate Institute of Medical Education and Research, Chandigarh, Chandigarh, IND

**Keywords:** eyelid tumor, masquerade, keratotic, basal cell carcinoma, cutaneous horn

## Abstract

Cutaneous horns are uncommon skin tumors consisting of keratotic material infrequently found on eyelids. We report the case of a 65-year-old male with a two-month history of cutaneous horn arising from the right lower eyelid. Histopathological examination following the excision biopsy disclosed a keratotic basal cell carcinoma (BCC). Basal cell carcinomas are slow-growing lesions with a history ranging from months to years. The keratotic form of basal cell carcinoma is a less common presentation of a cutaneous horn. A cutaneous horn is usually derived from an underlying lesion that may be benign, premalignant, or malignant. The diagnostic dexterity of keratotic BCC emphasizes the importance of histopathological confirmation in establishing the diagnosis and modifying management. To the best of our knowledge, this is the first case report of a keratotic basal cell carcinoma masquerading as a small cutaneous horn with such a short duration.

## Introduction

A cutaneous horn is an uncommon tumor that typically develops on sun-exposed skin in elderly individuals [1]. It comprises 4% of all eyelid tumors. It is composed of keratotic material [2]. They have a distinct appearance, resembling an animal horn. The underlying pathology with which the cutaneous horn is associated is difficult to diagnose clinically which may be benign, premalignant, or malignant. Recognizing these diagnostic dilemmas is important, thus emphasizing the need for early surgery with complete excision of the base. Therefore, histopathological examination of the base of the lesion remains vital to rule out a malignant lesion [3]. To our knowledge, the present case of keratotic basal cell carcinoma underneath a cutaneous horn has the shortest duration history ever reported in the literature.

## Case presentation

**Figure 1:**
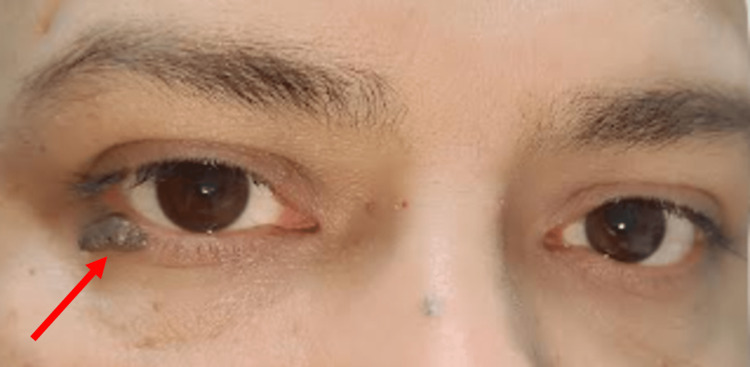
Patient image with the arrow pointing towards the right lower eyelid cutaneous horn

A 65-year-old male presented to the ophthalmology department with the chief complaint of a painless, growing conical mass on the right lower eyelid for the past two months. The patient was healthy with an unremarkable medical history. There was no history of prior cutaneous or systemic malignancies. Ophthalmological clinical examination demonstrated a firm horn-like lesion, 7 mm in height and 3 mm wide at the base (Figure 1). The lesion was pedunculated with a hyperkeratotic surface without the involvement of adnexal structures. The ocular examination was unremarkable.

**Figure 2:**
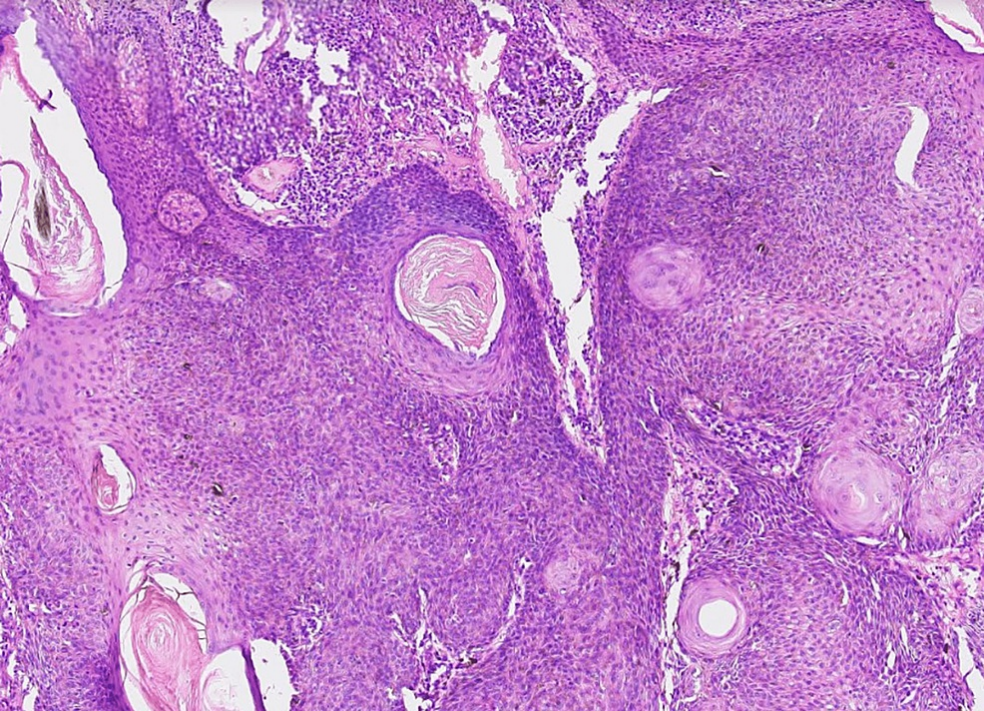
Biopsy shows an invasive tumor, arranged in islands composed of basaloid cells, with multiple keratin pearl formation

There were no systemic illnesses, with no history of either tobacco or cigarette smoking. There was no regional lymphadenopathy. The clinical diagnosis made was a solitary right lower eyelid cutaneous horn. The tumor was completely excised, and the primary closure of the defect was done with absorbable sutures. Histopathological examination confirmed an invasive keratotic basal cell carcinoma underlying the keratin horn. The tumor cells were arranged in islands and trabeculae. Within the islands and nodules, the tumor cells showed peripheral palisading with moderate nuclear pleomorphism, coarse chromatin, and an inconspicuous and moderate amount of cytoplasm. Central squamous differentiation in the form of keratin pearl formation was seen as shown in Figure 2. The wound healed without complications, and there has been no recurrence during the one-year follow-up.

## Discussion

A cutaneous horn is a morphological designation given to keratinized epidermal growths that can have spectacular dimensions and shapes. The associated etiologies are multiple and of variable nature. The cutaneous horn represents the tip of the iceberg. The base of the cutaneous horn may mask multiple conditions, including viral warts, actinic keratosis, keratoacanthoma, basal cell carcinoma, Bowen's disease, seborrheic keratosis, histiocytoma, pyogenic granuloma, follicular keratosis, discoid lupus erythematosus, verrucous epidermal nevus, molluscum contagiosum, and squamous cell carcinoma [2]. In a retrospective analysis of 48 cutaneous horns performed by Mencía-Gutiérrez, et al. from 1998 to 2002, histologically, 77.1% were benign, 14.6% were premalignant, and 8.3% were caused by malignant skin tumors. All malignant lesions were large 1.29 cm X 0.85 cm in size [2]. Table 1 highlights previous studies that have described the cornu cutaneum of the eyelid with underlying basal cell carcinoma.

**Table 1:** Previous studies that have described cornu cutaneum of the eyelid with underlying basal cell carcinoma

S No	Authors	Year	Number of patients	Age	Gender	Laterality	Description
1	Mencía-Gutiérrez E et al. [2]	2004	Two	< 40 years, 40–70 years	Not available	Not available	Not available
2	Tambe K et al. [4]	2012	One	65 years	Female	Right lower eyelid	Gradually progressing lesion for nine months, 3 mm base X 4 mm height
3	Foley P et al. [5]	1995	One	62 years	Female	Left upper eyelid	Gradually progressing lesion for one year

Cutaneous horns on histopathology show an abundance of compact keratin protruding from the epidermis. A plethora of epidermal lesions, ranging from benign to malignant epidermal proliferation, give rise to the basal layer of the epidermis in the cutaneous horn. Thus, the characteristic features of the pathologic process responsible for the cutaneous horn are exhibited by the base. Hence, histopathology remains primordial in any cutaneous horn. The type of lesion and its malignant potential will guide further management. An excisional biopsy of the lesion, including the base, followed by a histopathological examination to rule out malignancy is obligatory. In our patient, the cutaneous horn was masking keratotic basal cell carcinomas (BCC), which is a well-described but uncommon variant of BCC. The shortest presenting duration of basal cell carcinoma with overlying cutaneous horn reported in the literature is nine months [4], as compared to the two-month duration in our case, which is the shortest duration reported till now. Histologically, a keratotic BCC shows islands of basaloid cells with nuclei demonstrating peripheral palisading, similar to the solid or nodular form of BCC. However, squamous differentiation and the presence of keratinization in the center of the islands are its differentiating features [5].

## Conclusions

The cutaneous horn of the eyelid is a clinical diagnosis masking numerous lesions, therefore necessitating histopathological confirmation. The notion that large cutaneous horns are derived from a malignant base is debatable. Even a small cutaneous horn with a short history may be masquerading as an underlying malignancy. We conclude that all patients presenting with cutaneous horn should be operated on at the earliest as it may be overlying a malignant lesion like keratotic basal cell carcinoma.
